# Abdominal mass hiding rib osteomyelitis

**DOI:** 10.1186/s13052-016-0251-x

**Published:** 2016-04-12

**Authors:** Genny Raffaeli, Irene Borzani, Raffaella Pinzani, Caterina Giannitto, Nicola Principi, Susanna Esposito

**Affiliations:** Pediatric Highly Intensive Care Unit, Department of Pathophysiology and Transplantation, Università degli Studi di Milano, Fondazione IRCCS Ca’ Granda Ospedale Maggiore Policlinico, Via Commenda 9, 20122 Milan, Italy; Pediatric Radiology Unit, Fondazione IRCCS Ca’ Granda Ospedale Maggiore Policlinico, Milan, Italy; Radiology Unit, Fondazione IRCCS Ca’ Granda Ospedale Maggiore Policlinico, Milan, Italy

**Keywords:** Bone infection, MRSA, Osteomyelitis, Rib, *Staphylococcus aureus*

## Abstract

**Background:**

Rib osteomyelitis is a rare entity, occurring in approximately 1 % or less of all cases of haematogenous osteomyelitis. Given its rarity and clinical heterogeneity, the diagnosis of rib osteomyelitis can be challenging and requires a high index of suspicion. We present a case of acute osteomyelitis of the rib due to community-acquired methicillin-resistant *Staphylococcus aureus* (MRSA), which occurred in an otherwise healthy 3-month-old infant and mimicked an epigastric hernia at first.

**Case presentation:**

An otherwise healthy 3-month-old female infant was sent by her primary care paediatrician to the paediatric emergency department for possible incarcerated epigastric hernia because for 2 days, she had suffered from mild to moderate fever, irritability, poor feeding, and tender epigastric swelling. Ultrasonographic imaging excluded epigastric hernia, and transthoracic echocardiography ruled out endocarditis. However, clinical assessment combined with laboratory criteria classified the child into the high-risk group for having severe bacterial infection. Consequently, awaiting the definitive diagnosis, she was immediately treated with a broad-spectrum regimen of intravenous antibiotic therapy based on vancomycin (40 mg/kg/die in 3 doses) and meropenem (100 mg/kg/die in 3 doses). Three days after admission, the blood culture result was positive for methicillin-resistant *Staphylococcus aureus*, and vancomycin remained as antibiotic therapy. On day 3, a second swelling appeared at the level of the seventh left rib, 2 cm-wide, non-erythematous, mildly painful. Ultrasonography of the left chest wall on this occasion showed an image consistent with an acute osteomyelitis of the anterior osteo-chondral region of the 7th rib and associated adjacent periosteal and soft tissue collection and magnetic resonance imaging confirmed the osteomyelitis of the anterior middle-distal part of the 7th left rib, near the costochondral junction. Vancomycin was continued up to a total of 6 weeks of therapy, and at the end, the child was discharged in good condition with no relapse during the follow-up.

**Conclusion:**

This is one of the few reported cases of paediatric rib osteomyelitis caused by community-acquired MRSA. Timely identification associated with prompt and targeted antibiotic therapy may allow full recovery.

## Background

In industrialized countries, acute osteomyelitis occurs in approximately 8 per 100,000 children per year. In most cases, particularly in younger children, it results from haematogenous spreading [1]. Generally, osteomyelitis occurs in the metaphysis of long bones. Involvement of short bones is rare. Rib osteomyelitis accounts for no more than 1 % of all the haematogenous osteomyelitis cases [2]. In most children, the disease presents with fever accompanied by a mass in the chest, anteriorly, near the costochondral junction, or posteriorly, near the costovertebral angle. However, sometimes, as in the case reported here, local signs and symptoms are mild, and no evidence of local infection is present, leading to wrong suppositions [3], such as the diagnosis of possible epigastric hernia. We present a case of acute osteomyelitis of the rib due to community-acquired methicillin-resistant *Staphylococcus aureus* (MRSA), which occurred in an otherwise healthy 3-month-old infant and mimicked an epigastric hernia at first.

## Case presentation

An otherwise healthy three-month-old female infant was sent by her primary care paediatrician to the paediatric emergency department for possible incarcerated epigastric hernia because for 2 days, she had suffered from mild to moderate fever, irritability, poor feeding, and tender epigastric swelling. The infant was born at term after an uneventful pregnancy with no maternal infection during pregnancy, with a normal delivery, without any local trauma, with weight and length within normal limits and an Apgar score of 9/10. Her past medical history was unremarkable. Any local trauma was denied. On admission, the infant was found to be pale, febrile (axillary temperature, 39 °C), irritable, lethargic, and feeding poorly. Examination revealed a slightly erythematous soft swelling of 2 × 3 cm, mildly painful by palpation, involving the epigastric area without any inflammatory local signs. The rest of her physical examination was unremarkable.

At admission, relevant laboratory findings included decreased haemoglobin concentration (6.8 g/dL; normal value, ≥13.5 g/dL), increased white blood cell count (32,000 cells/mm^3^; normal value, 10,000-25,000 cells/mm^3^) with 65 % neutrophils, and increased C-reactive protein (17.4 mg/dL; normal value, <4 mg/dL), procalcitonin (1.2 ng/mL; normal value, <0.01 ng/mL), and erythrocyte sedimentation rate (38 mm/h; normal value <10 mm/h). Chest and abdominal radiographs were normal. Ultrasonographic imaging excluded epigastric hernia, and transthoracic echocardiography ruled out endocarditis.

Clinical assessment combined with laboratory criteria classified the child into the high-risk group for having severe bacterial infection. Consequently, awaiting the definitive diagnosis, she was immediately treated with a broad-spectrum regimen of intravenous antibiotic therapy based on vancomycin (40 mg/kg/die in 3 doses) and meropenem (100 mg/kg/die in 3 doses). Three days after admission, the blood culture result was positive for methicillin-resistant *Staphylococcus aureus,* and vancomycin remained as antibiotic therapy.

**Fig. 1:**
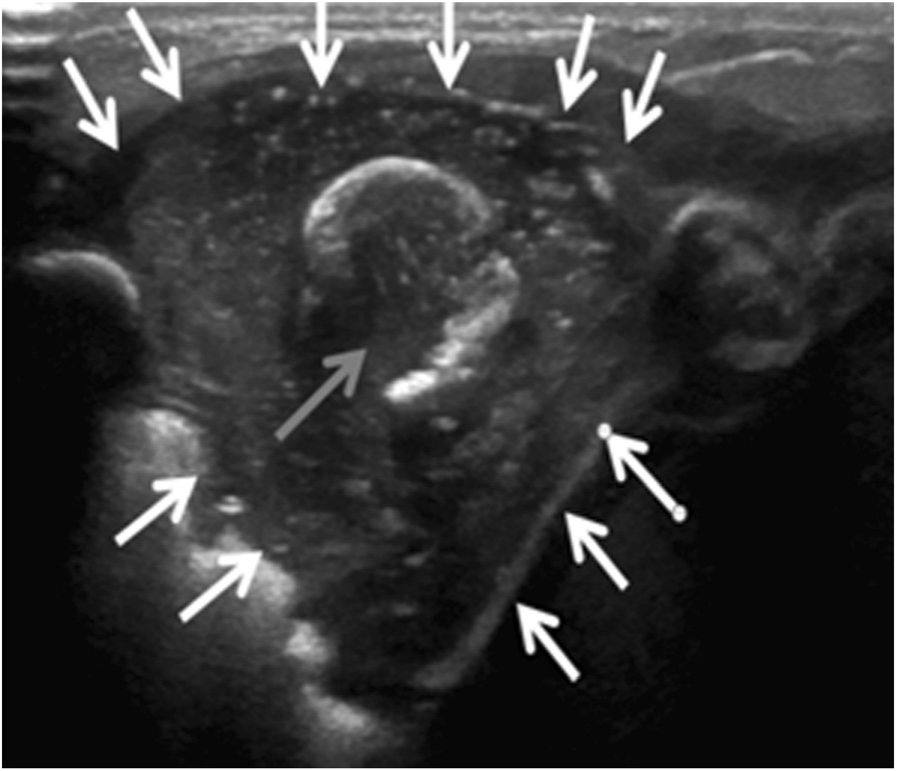
Ultrasonography. There is a hypoechoic fluid collection (white arrows), related to an abscess, encasing a fragmented rib (grey arrow)

**Fig. 2:**
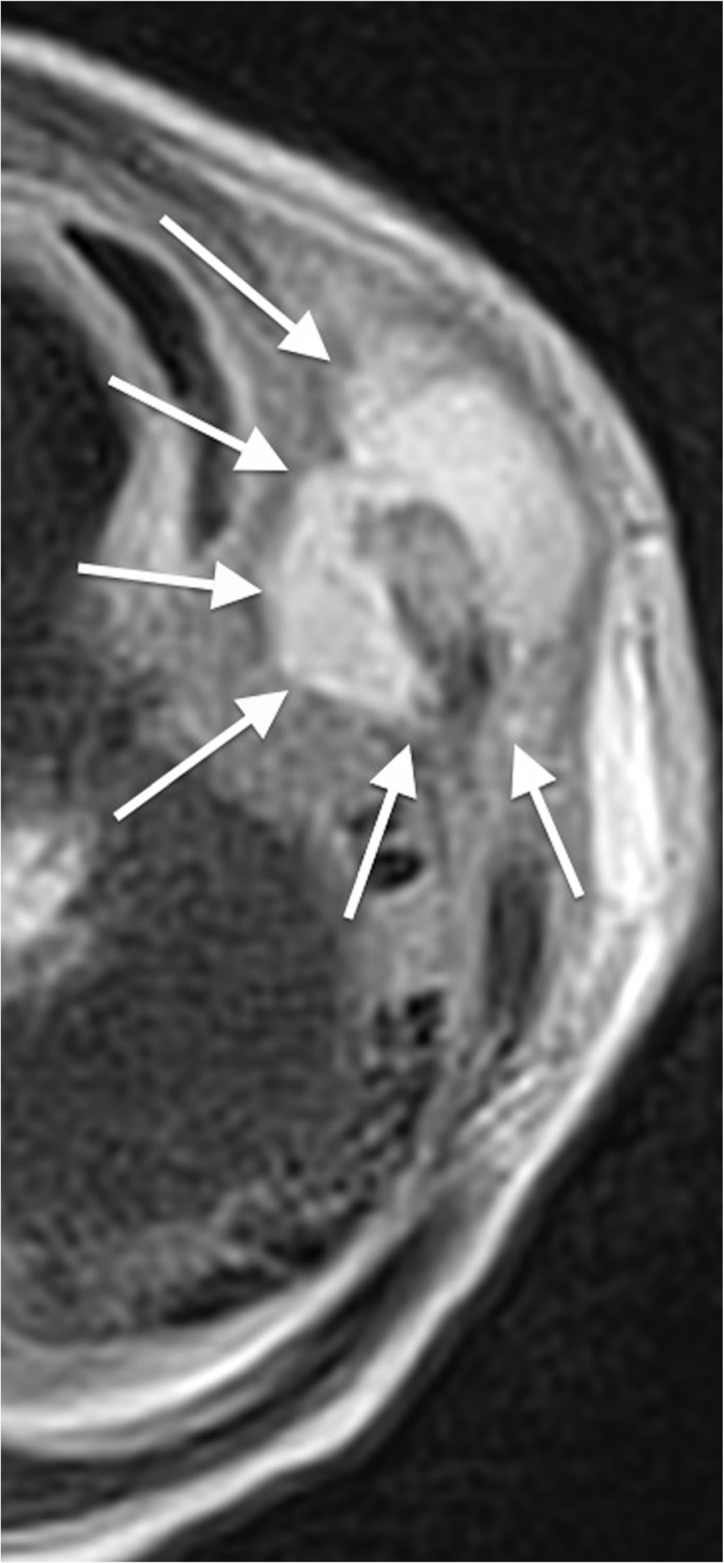
Rib osteomyelitis: magnetic resonance imaging. The axial T2-weighted image shows a fluid collection with high signal intensity, encasing the anterior portion of the seventh left rib (white arrows). The rib is deformed and fragmented, with bone marrow hyperintensity

On day three, a second swelling appeared at the level of the seventh left rib, 2 cm-wide, non-erythematous, mildly painful. Ultrasonography of the left chest wall on this occasion showed an image consistent with an acute osteomyelitis of the anterior osteo-chondral region of the 7th rib and associated adjacent periosteal and soft tissue collection (Fig. 1). Magnetic resonance imaging (MRI) confirmed the osteomyelitis of the anterior middle-distal part of the 7th left rib, near the costochondral junction (Fig. 2). It also revealed intra-thoracic involvement with a small amount of pleural effusion and localized parenchymal consolidation. In epigastrium, a 32 × 19-mm mass was documented, impacting the anterior margin of the liver. Adjacent to this, two additional flogistic intrahepatic areas (approximately 2 cm-wide and 2.5 cm-wide) were described. No other focal alteration was observed in the contest of the other intra-abdominal organs.

During the hospital stay, the infant gradually improved, and the laboratory parameters returned to normal values within two weeks. No surgical drainage of the rib was required.

After four weeks of medical therapy, follow-up MRI showed general improvement with a marked dimensional decrease of the osteo-chondral lesion of the 7th rib (9 × 19 mm vs 22 × 25 mm) and complete resolution of the epigastric abscess. Intrahepatic alterations were still visible, although smaller than previously reported (1.3 cm-wide and 0.8 cm-wide, respectively), whereas the pleural effusion resolved completely.

The patient did not experience any MRSA-related complications during the hospital stay. Vancomycin was continued up to a total of 6 weeks of therapy, and at the end, the child was discharged in good condition. A chest radiograph obtained two weeks after discharge showed a widening and a sclerosis of the previously affected bone, consistent with a late effect of the preceding infection. A concomitant sonographic examination revealed complete resolution of the intra-abdominal abscessual lesions.

The child recovered very well, presenting a hard swelling at the level of the 7th rib, which was not painful and was stable over time, as a plausible remnant of the previous infection. In serial follow-up assessments, after three months, no relapse of osteomyelitis was observed.

## Conclusions

Our case shows that rib osteomyelitis can be easily misdiagnosed because of its rarity and non-specific clinical signs. However, paediatricians should be aware of its possible clinical manifestations, maintaining a high index of suspicion. Timely identification associated with prompt and targeted antibiotic therapy may allow full recovery. Awareness of the increasing community-acquired MRSA infection rates may help to optimize therapeutic strategies.

Because of its rarity and because the earliest signs and symptoms of the disease can be subtle and nonspecific, diagnosis and adequate treatment in rib osteomyelitis are frequently delayed. In most reported cases, the diagnosis exceeded a 6-month period, leading to the need for surgical treatment to cure the patient. Even in atypical cases, early diagnosis is favoured by the use of modern methods of diagnostic imaging [4–6]. As evidenced by this case, ultrasound scanning appears to be a valuable diagnostic tool for the first evaluation of soft-tissue changes related to bone infection [7, 8]. It reveals pericostal oedema, which, although not specific for osteomyelitis, can be evocative, particularly when associated with suggestive clinical signs and leading to MRI, which remains the most sensitive and specific diagnostic test [4–6].

From an etiologic point of view, contrary to what happens in the developing world where rib osteomyelitis is frequently due to *Mycobacterium tuberculosis* [2], in children living in industrialized countries, this disease is mainly due to *S. aureus.* This case is not an exception. However, a MRSA was evidenced as the cause of the disease. Because the child was never hospitalized and because since birth she did not suffer from any infectious disease requiring antibiotic treatment, it is highly likely that it was a community acquired-strain. This case highlights the increasing importance of community-acquired MRSA in the aetiology of osteomyelitis [9] and the need to include drugs active against this particular pathogen, such as vancomycin, in the initial treatment of paediatric osteomyelitis with later substitution with a penicillinase-resistant betalactam antibiotic if methicillin-resistant strains are evidenced [10].

## Consent

This case report has been approved by the Ethics Committee of Fondazione IRCCS Ca’ Granda Ospedale Maggiore Policlinico, Milan, Italy. Written informed consent was obtained from the patient’s parents for publication of this case report. A copy of the written consent is available for review from the Editor-in-Chief of this journal.

## Abbreviations

MRI: magnetic resonance imaging
MRSA: methicillin-resistant *Staphylococcus aureus*
